# Improved insights into the SABIO-RK database via visualization

**DOI:** 10.1093/database/baad011

**Published:** 2023-03-31

**Authors:** Dorotea Dudaš, Ulrike Wittig, Maja Rey, Andreas Weidemann, Wolfgang Müller

**Affiliations:** Scientific Databases and Visualization, Heidelberg Institute for Theoretical Studies (HITS gGmbH), Schloss-Wolfsbrunnenweg 35, Heidelberg 69118, Germany; Scientific Databases and Visualization, Heidelberg Institute for Theoretical Studies (HITS gGmbH), Schloss-Wolfsbrunnenweg 35, Heidelberg 69118, Germany; Scientific Databases and Visualization, Heidelberg Institute for Theoretical Studies (HITS gGmbH), Schloss-Wolfsbrunnenweg 35, Heidelberg 69118, Germany; Scientific Databases and Visualization, Heidelberg Institute for Theoretical Studies (HITS gGmbH), Schloss-Wolfsbrunnenweg 35, Heidelberg 69118, Germany; Scientific Databases and Visualization, Heidelberg Institute for Theoretical Studies (HITS gGmbH), Schloss-Wolfsbrunnenweg 35, Heidelberg 69118, Germany

## Abstract

SABIO-RK is a database for biochemical reactions and their kinetics. Data in SABIO-RK are inherently multidimensional and complex. The complex relationships between the data are often difficult to follow or even not represented when using standard tabular views. With an increasing number of data points the mismatch between tables and insights becomes more obvious, and getting an overview of the data becomes harder. Such complex data benefit from being presented using specially adapted visual tools. Visualization is a natural and user-friendly way to quickly get an overview of the data and to detect clusters and outliers. Here, we describe the implementation of a variety of visualization concepts into a common interface within the SABIO-RK biochemical reaction kinetics database. For that purpose, we use a heat map, parallel coordinates and scatter plots to allow the interactive visual exploration of general entry-based information of biochemical reactions and specific kinetic parameter values.

**Database URL** https://sabiork.h-its.org/

## Introduction

SABIO-RK (https://sabiork.h-its.org/) is a manually curated database for biochemical reactions and their kinetic properties (1). Each dataset (‘database entry’) is describing an experimental result, i.e. kinetic measurements of a biochemical reaction in the context of the entire experimental set-up including details about reaction participants and the origin of the catalyzing enzyme.

Data are mainly manually extracted from the literature including publications from the 1960s to present. When entering data into SABIO-RK, correctness is evaluated with respect to the original publication, but there is no additional evaluation concerning correctness of the measurement or quality of the biological or experimental set-up. As a result, kinetic parameters for the same biological source and the same experimental set-up can vary and strongly differ from each other, just as the underlying publications. The SABIO-RK database curators’ work is based on the information given in the publication. Consistent with this policy, all relevant data in a publication are inserted into SABIO-RK and always point to the original source.

After >15 years of data insertion into SABIO-RK with >300 000 kinetic parameters extracted from ∼7500 publications, the database has now reached a quality and quantity that makes the visualization of data interesting and worthwhile.

Biological databases containing kinetic parameters mostly show their search results in table or text-like formats (e.g. UniProtKB (2) and BRENDA (3)). They have many tools to represent the protein structure and genomic data visually but no interactive tools for representing the kinetic information.

The BRENDA enzyme database allows the user to visualize and compare the full value distribution of numerical parameters with ‘the functional parameter statistics’ (4), which is useful to get an idea whether a measured value is comparable to others or should be considered as an outlier. This visualization is, however, not interactive and cannot be used to define a database query in more detail.

Our aim for the SABIO-RK database is to introduce visual navigation through the search results by taking advantage of the natural human affinity to easily interpret visually presented data. By definition, visualization is the use of computer-supported, interactive, visual representation of data to amplify cognition (acquisition or use of knowledge) (5). Data visualization is especially an essential tool for revealing insights buried in complex data (6). Since a single database entry of SABIO-RK is complex and contains >30 attributes, such a visualization would help the user to get an overview about the context of the attribute of interest and to refine the search easily.

Since the focus of SABIO-RK is on biochemical reaction kinetics, the goal of the visualization project is to get a better overview about the data in SABIO-RK and to get an idea about the dimension of the kinetic parameter distribution. The visualization of the distribution of parameters for a specific query allows the detection of clusters and outliers or surprising categorical values from a list of parameters and, for example, helps modelers to select the best values for model creation in the context of the adequate experimental conditions.

## Methods

Originally, SABIO-RK data are searchable in the user interface using a free-text query or advanced search functionality and limited visual search using a bar chart search. Research results are given in sortable overview tables in Entry View and Reaction View as well as in a bar chart search that allows to refine the search by choosing only one organism, tissue, cell location, EC number, UniprotID, kinetic parameter type or kinetic law type. The table format contains only a subset of data, and the full dataset of a database entry can be expanded to view the additional textual information. Textual formats, the table and the entry format, are not able to give a complete summary of the query result. Here, we present a visualization of the search results in SABIO-RK, which overcomes the limitations of the previously mentioned result view formats.

### SABIO-RK software considerations

SABIO-RK is somewhat typical small-team software that has been developed over the years with tight timing constraints and tight constraints on continuous availability. One side condition of such software is that adding layers is preferable to deep refactoring that may break compatibility. As a consequence, the visualization was added as a layer on top of SABIO-RK with minimal changes to the underlying SABIO-RK. Due to such considerations, the visualization was developed by abiding by various constraints and by overcoming different data structure challenges.

The SABIO-RK web interface was implemented using a Grails/Groovy framework and both the PostgreSQL database and the Apache Solr search engine at the backend. The visualization was implemented using JavaScript and D3.js v5 and embedded in Groovy server pages.

A database entry in SABIO-RK is defined as one single reaction, including the reaction participants, measured in a specific organism under specific environmental conditions. For one single database entry, more than one kinetic parameter (e.g. kcat, Km and substrate concentration) can be given. As a search engine, Apache Solr is used. A single database entry in the SABIO-RK database is represented as a single object in the Solr index with its attributes. Because one entry has many kinetic parameters, a parent–child relationship was used for the storage of the kinetic parameters for each entry. Queries are submitted by the client as asynchronous requests to a SABIO-RK controller class, and the results of the following Solr query are returned in JSON format. Each entry and each kinetic parameter are returned as separate objects, with kinetic parameters containing the EntryID attribute of the entry they belong to. Since one entry can have several kinetic parameters, it is useful to have graphs representing both entities. Based on the separate handling of kinetic parameters in the database, two additional representations dealing with kinetic parameters are added to the entry-based visualizations.

The speed and usability of the new visualization were repeatedly tested and improved. Some computing constraints were also needed to accommodate showing large amounts of data in a web browser. The maximum number of resulting data entries that can be shown is limited and set to 10 000. This limit only applies for the visualization and was set through trial and error by finding a compromise between the time needed to show the data on the graphs, graph clutter and getting browser issues. Note that an arbitrary number of data can be exported from the SABIO-RK database independent of the visualization. Although showing a larger number of results in the visualization is possible, users do not want to wait for a very long time for these results to be loaded and drawn in the graphs. The amount of data that is by default shown in parallel coordinates and the scatter plot matrix is limited to 400 in order to avoid the graph clutter and longer loading time. The ‘Allow More Data’ checkbox appears if the number of results is between 400 and 10 000 and allows overriding this limitation. The initial limitation to only show up to 10 000 entries is hard-coded and cannot be overridden.

Multiple user tests were made testing the usability and functionality of the visualization and user comments and requests considered or implemented. In addition, several online workshops were held as a part of the de.NBI training courses ‘Tools for Systems biology modeling and data exchange: COPASI, CellNetAnalyzer, SABIO-RK, FAIRDOMHub/SEEK’ to train users and test the visualization. The user feedback was generally positive with a high learning curve: once time was taken to shortly learn about the usage of the graphs, the users were capable of solving complex tasks without the supervision and found the offered visualizations to be very useful.

## Results

An additional tab labeled ‘Visual Search’ was added to the SABIO-RK user interface to facilitate searching through the data and add an alternative way of looking at the data collected within the SABIO-RK database. It offers a graphical interface to explore the SABIO-RK database. Instead of composing a complex text query, the SABIO-RK database can now be navigated by using different implemented graphs.

**Figure 1.**
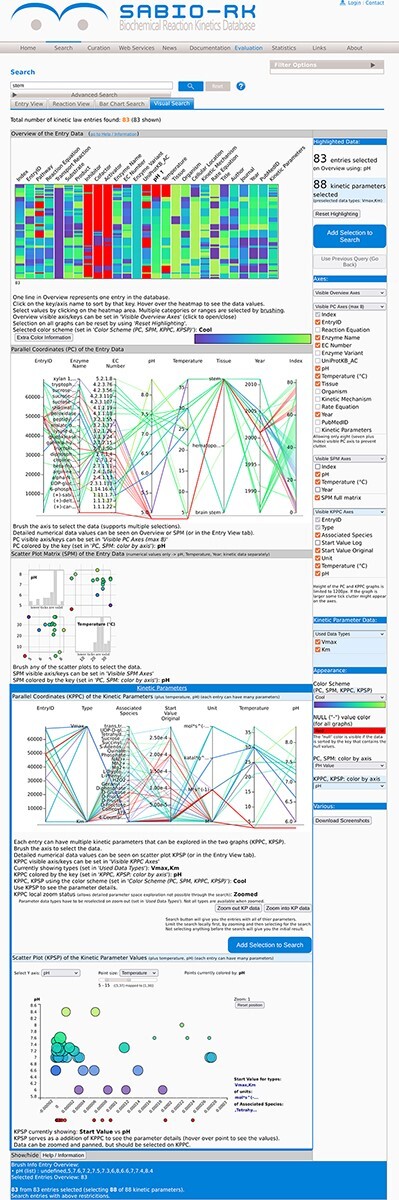
The visualization module of the SABIO-RK database. First three graphs (the heat map overview, the parallel coordinates and the scatter plot matrix with histograms) are representing the entry-based graphs. Bottom two framed graphs are kinetic parameters–based graphs.

After the initial search term is set in the text field, the Entry View tab of the web interface shows the information about the resulting entries by default. Switching to the Visual Search tab shows the new visualization module (Figure 1).

### Entry-based graphs

There are three different visualization concepts used in the new SABIO-RK visualization to represent the data: (i) heat map overview, (ii) parallel coordinates and (iii) scatter plots. Clicking on the Visual Search tab of the SABIO-RK interface, an overview heat map graph of the search result and (depending on the number of the results) parallel coordinates graphs and scatter plots with histograms (Figure 1) are displayed.

**Figure 2.**
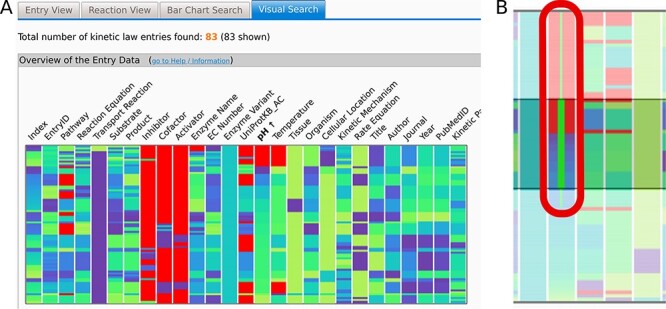
(A) The Overview heat map graph. Attribute names are given above the graph. Data can be sorted by any of the attributes. Here, the data are sorted by pH values. One horizontal line represents one entry of the data. Data can be selected by clicking or brushing. (B) Brushing extends the selection to capture all of the brushed border values (thin green line in (B)) including all entries with the same highlighted property (e.g. pH value 7.0). Selected data are highlighted in all of the graphs. Axes can be added or removed and rearranged as desired as well as the used color schemes. Here, the null values are denoted in red, while a ‘cool’ color scheme ranging from dark purple to lime is used per attribute or axis.

The overview graph (Figure 2A) is a heat map implemented using the Navio (7) JavaScript library and adjusted and expanded according to the needs of the SABIO-RK data and the requests based on feedback from the curators and the users. It lists the SABIO-RK search attribute names above the column representing the color-coded values of the attribute in question (e.g. Temperature and Enzyme Name). The data value is visible in a tooltip when hovering above the value with a mouse pointer. Data can be sorted by any of the attributes when clicking on the attribute name. One horizontal line represents one entry of the data and its different values. All graphs are interactive, and selecting data on one graph selects the same data on the other graphs. Data in the overview graph can be selected by clicking or brushing. Brushing extends the selection to capture all of the brushed border values (represented by the thin green line, see Figure 2B) including all entries with the same highlighted property (e.g. pH value 7.0).

Users can adjust what is being viewed (choose the attributes) and how it is being viewed on any of the graphs by selecting options in the user interface or directly on the graphs. The axes in the overview graph can be added or removed and rearranged as desired, and the used color schemes can be changed with the color for the null values specifically chosen. Null values are the values where no information is given for a specific entity within a database entry due to missing information in the publication of origin.

**Figure 3.**
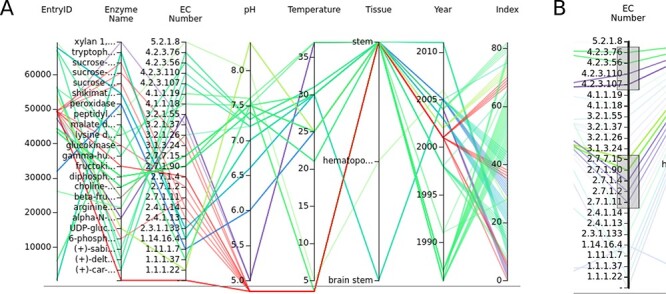
(A) The parallel coordinates graph of the entry data. Attribute names are given above the graph. One entry represents one line on the graph. Null values are under the lower line. (B) Data can be selected by brushing the axes.

Parallel coordinates is another way to visualize the multidimensional data of the SABIO-RK. It shows different data attributes with its values on the parallel axes connected by a line. In entry-based parallel coordinates, one line in the graphs represents one entry of the SABIO-RK data. Attribute names are given above the graph and can be used to rearrange the axes of the graph. Null values are shown under the lower line (Figure 3A). Data on parallel coordinates can be selected by brushing the axis, i.e. dragging the mouse pointer over a selection above the axis area (axes get highlighted when it is possible to select them and are bold if something is selected on them). It is also possible to have several separated selections (Figure 3B). If the axis ticks appear too crowded (graph adjusts its height to the data but also has a limited height), one can select and zoom into the data by doing the search.

**Figure 4.**
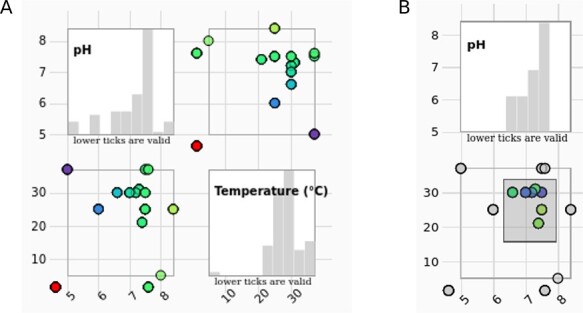
(A) The scatter plot matrix of the entry data with histograms is showing measurement conditions (pH values in correlation with temperature) of the selected entries. (B) The data range can be selected by brushing on any of the scatter plots. Histograms change accordingly.

The scatter plot matrix (Figure 4A) is a collection of the standard point graphs for the numerical attributes of the SABIO-RK data (pH, temperature). The diagonal graphs in the scatter plot matrix are showing the histogram values of the attribute in question (instead of showing the diagonal points, e.g. pH vs pH). The range of data can be selected by brushing within any of the scatter plots (Figure 4B). Histograms change accordingly.

### Kinetic parameter–based graphs

The three entry-based graphs were extended by additional graphs to show kinetic parameters, i.e. the kinetic data of the entries (e.g. Km, Vmax and kcat). Unlike the first three graphs indexed by the database entries (meaning one entity on the graph—a line or a point—is representing one entry of the database), the last two graphs are kinetic parameter–based graphs, namely (i) a separate parallel coordinates graph and (ii) a scatter plot graph. Since each entry can contain several kinetic parameters with its types, values, units and associated species, these parameters are dealt with separately, allowing for better exploration of the kinetic data space and its connections to the rest of the data in SABIO-RK.

When doing the search query in SABIO-RK, the result is a number of entries including all of their kinetic parameters. It is not possible to separately explore the kinetic parameters space when dealing with whole entries. By utilizing the parallel coordinates plot based on the kinetic parameters, it is possible to locally move (zoom in/out) through the kinetic parameter data space. Prior to searching the whole database for the entire entries containing our selection of the kinetic data (as noted, the query search gives all of the kinetic parameters of the resulting entries), the user can locally zoom in and zoom out of the kinetic parameter data space, which allows for a more precise composition of the wanted search. This makes it possible to fully explore the kinetic parameter data space since this is not doable using the text search queries due to the lack of keywords dealing with the kinetic parameters (see the section ‘SABIO-RK Software Considerations’). For example, searching for the range of the kinetic parameter values is not possible directly by using the text query, but, after the user selects such a range in the parallel coordinates, it is indirectly made possible by using the EntryIDs of the selected range. The resulting search then gives entries and all of their parameters as the result.

**Figure 5.**
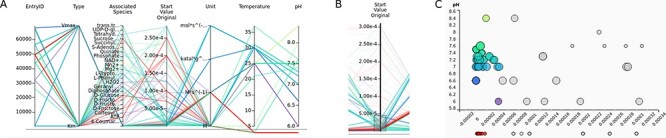
(A) Parallel coordinates of the kinetic parameters. (B) Data can be selected by brushing the axes. This selection automatically selects the data on the scatter plot. (C) The scatter plot helps to keep an overview of the exact values of the selected kinetic parameters.

Kinetic parameters are shown on an additional parallel coordinates plot indexed by the kinetic parameters themselves (Figure 5A). The user can explore the parameter space, similar to the entry data space, by using the kinetic parameters data attributes (Type, Associated Species, Value, Unit) represented as axes of the parallel coordinates. For convenience reasons, it is also possible to view the temperature and pH values also on this graph. The additional parallel coordinates plot is connected to the previous entry-based plots, and selecting data there means the corresponding entries are highlighted on the entry-based plots (heat map overview, parallel coordinates and the scatter plot matrix) and vice versa.

A preselection of the kinetic parameter data types shown in the graph is made, and only the most prominent types are shown in the beginning (Km, Vmax, kcat); however, the user can choose which types should be shown in the right-side graphical user interface (GUI).

**Figure 6.**
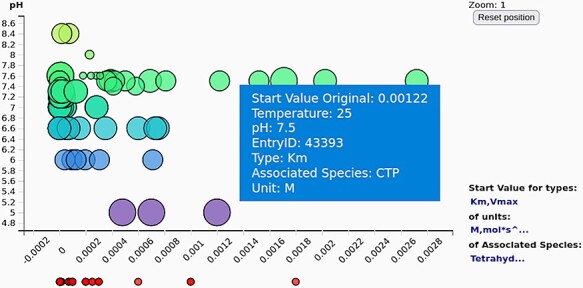
The scatter plot of the kinetic parameter values (here shown against the pH values) gives an overview of the exact values of the selected kinetic parameters (often not deductible from the axis tick denotations on the parallel coordinates).

An additional scatter plot (Figure 5C and Figure 6) is introduced to show the connection of the kinetic parameter values and a chosen numerical attribute (can be temperature, pH value or simply entry id) and allow the user to precisely view the values of the parameters shown within the tooltip when hovering above the point using the pointer. Note that precise parameter values are often not deductible on the parallel coordinates because of the axis label denotations on the numerical axes that are not explicitly listing the data value. Data cannot be selected on this scatter plot. This graph responds to selection on the parallel plot of the kinetic parameters where users can select wanted types, units and associated species of the data (Figure 5B and C). Null values of the chosen attribute (pH or temperature) are shown under the graph area. The scatter plot can show the third value as a size of the point, making it effectively a 2.5D graph (with an imprecise third dimension). The user can zoom in and out of the points shown in the scatter plot by using the mouse wheel.

By combining the two kinetic data graphs, it is possible to gain a better insight into the kinetic data at hand. These graphs can be used by regular users as well as by curators to identify clusters or outliers of kinetic parameter values or even errors in the data.

### Searching the data and parsing the search query

**Figure 7.**
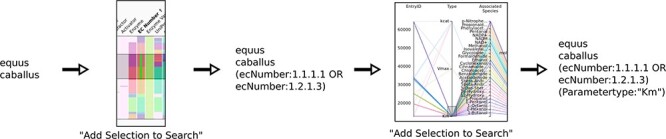
Schematics of searching using the new visualization and query composition for the SABIO-RK data.

When data are selected on one of the graphs and the ‘Add Selection to Search’ button is pressed, a text search query is composed and added to the search text area of the SABIO-RK web interface (Figure 7).

The created query differentiates between the categorical and numerical data attributes. Numerical data use the ranges, resulting in parts of the query containing [X TO Y]. Categorical data are listed, resulting in the queries of the type A OR B OR C etc. Special characters (e.g. reaction equation values usually contain ‘+’) and brackets have to be dealt with to keep the query parsable. The queries are not intelligently reparsed on every creation, and there is no removal of the duplicate logical conditions when zooming into the data, i.e. when refining the query. The query is simply refined by adding the new condition on the top (or rather to the end) of the existing conditions (Figure 7). This makes it possible to exactly determine a single step of the search by examining the query text as the new constraints are added. It is possible to ‘undo’ the newest data search and revert to the previous search query by pressing ‘Use Previous Query (Go Back)’ in the user interface. Dealing with queries made using the kinetic parameter parallel coordinates graph has special limitations. Certain kinetic parameter attributes cannot be used directly in the search query, e.g. the start parameter value, where it is not possible to directly search for the start value range. This is handled so that the user can choose the value range on the kinetic parameter parallel coordinates, but the query is then formulated by listing the EntryIDs to which the kinetic parameters belong to.

### Customizing the graphs

**Figure 8.**
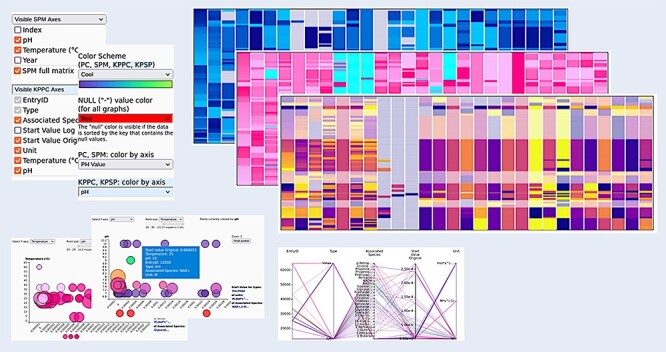
Customizing the graphs. All graphs can be visually adjusted by determining what exactly is shown within the graphs (choose the axes), by reordering the data and by selecting different color schemes for the visualizations, making the appearance adjustable to the needs of the user.

The right-side GUI can be used to set the desired properties of the graphs, such as which attributes are shown, or to select a different color scheme to better express the desired properties of the data (Figure 8). Created plots can be exported as PNG images in the GUI, and resulting data can be exported from the Entry tab.

The Overview heat map uses the entire color scheme for each of the attributes, i.e. columns stretched over the respective attribute range. Meaning, for example, that the EntryID attribute will show the full spectrum of the color scheme and the attribute, i.e. column containing only two values (e.g. Rate Equation), will show the two end values of the color scheme. All other graphs are colored by a single attribute that can be chosen in the user interface to better emphasize the desired properties of the data. This also happens automatically when sorting the values in the Overview by selecting an attribute (i.e. by clicking on its name in the Overview). The other entry-based graphs then get colored by the exact color scheme of that selected attribute. In the coloring sense, kinetic parameter–based graphs are independent of the entry-based graphs, except when dealing with pH and temperature attributes. Sorting the Overview by pH or temperature will color the kinetic parameter–based graphs by using the pH or temperature color range.

Details about the different graphs and features and options for customizing the visualization are given in a comprehensive help document. Additional video help files showing the usage of the visualization are going to further increase the usability of the visualization.

## Use cases

### Specific scientific query shows outlier values

**Figure 9.**
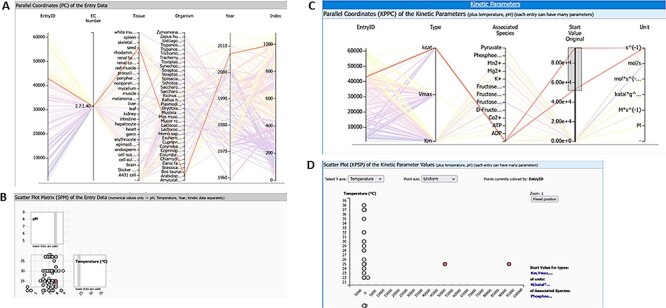
Query results for enzyme pyruvate kinase and reaction ‘ATP + Pyruvate = ADP + Phosphoenolpyruvate’ (query term: ECNumber:”2.7.1.40” AND SabioReactionID:”9”) with two highlighted kinetic parameter outlier values: (A) parallel coordinates of entry data, (B) scatter plot matrix of entry data, (C) parallel coordinates of kinetic parameters, and (D) scatter plot of kinetic parameters.

The SABIO-RK visualization enables a user-friendly way to find outliers for specific scientific questions. An example search for the enzyme pyruvate kinase (EC 2.7.1.40) and the corresponding reaction ‘ATP + Pyruvate = ADP + Phosphoenolpyruvate’ (query term: ECNumber:”2.7.1.40” AND SabioReactionID:”9”) gives a result of 1084 database entries in SABIO-RK. From an overview about the corresponding kinetic parameters, two kcat values (highlighted in Figure 9) seem to be out of the range of all other values (Figure 9C and D). The related scatter plot (Figure 9B) shows that the experimental conditions are in a cluster with the other values and with pH 8 and temperature 25°C not in the extreme range. Due to the interaction of the different graphs, highlighting the outlier values in the parallel coordinates graph of the kinetic parameters (Figure 9C) immediately gives a deeper insight into the entry data and the corresponding biological source (Figure 9A). Here, the example shows that the outlier values were measured in the organism *Arabidopsis thaliana*. The selection can be added to search in order to find more details in the database entries and to check the publication.

### Find perfect experimental condition for a given enzyme

The visualization can not only be used to find outliers of kinetic values as given in the first use case, but the database can also be easily visually checked for outliers in experimental conditions, e.g. temperature or pH. In this use case, the search is done for data of Mammalia measured at untypically low temperature or pH. Searching for all mammals (query term: Organism:”mammalia (NCBI)”) currently results in 27 012 entries. Since the maximal number of entries allowed for visualization is restricted to 10 000 entries due to its high computing load (see the section ‘SABIO-RK Software Considerations’), the search for outliers of temperature or pH can be first refined by using the slider in the ‘Filter Options’ drop menu next to the search field. Here, the search result can be restricted to a temperature or pH range, which is unexpected for mammals. In our example, we chose temperature between −10°C and +2°C, resulting in 14 database entries derived from six publications. Looking into the publications of origin confirmed the correctness of the values in the database in this case. Explanations given by the authors (if at all) for measurements under these non-physiological conditions were instability of the enzymes or substrates at higher temperatures. The user of SABIO-RK should decide whether kinetic values measured under these conditions should be taken into account or not. This use case shows how the different search functionalities can be easily combined to get a reasonable visualization of outliers.

### Finding curation errors

The visualization in SABIO-RK can also be used to find annotation errors arising during the data curation process. As an example, a query for tissue ‘liver (BTO)’ results in nearly 10 000 database entries. By inspecting the organism names in the Bar Chart Search or Visual Search tab, it is obvious that *Ascaris suum* (pork roundworm) does not have a liver itself. Looking into the publications of origin shows that the enzyme PEPCK (phosphoenolpyruvate carboxykinase) was isolated from *Ascaris* muscle instead (8). Such curation errors are easier to find by a visual overview of the data instead of looking through different pages of tabular data in the Entry View tab.

## Discussion

In order to facilitate interactive search and data refinement through the SABIO-RK data, a new visualization module was developed. Its goal is to improve the understanding of the database content and detect possible discrepancies between kinetic parameters from different publication sources.

Measured kinetic parameters published in the literature vary in magnitudes not only because of different experimental conditions (e.g. temperature, pH and buffer) but also because of different sensitivities of the measurements or other biological or experimental factors influencing the enzymatic reaction (e.g. unknown isozymes). An example of variances of parameter values for enzymes of the aerobic energy metabolism in yeast collected from different literature sources is given in Table 3 of a review article focusing on modeling (9). As shown in the ‘Use Cases’ section, our visualization of the parameter values helps to easily define the ranges of values, get an impression of clusters and find outliers.

The definition of outliers highly depends on the scientific question. A value could cluster within a set of parameters for a given organism but could be an outlier under specific experimental conditions. The combination of different search criteria can define a value as an outlier or not, and therefore for the evaluation, an overview of all related data is important.

In addition, the quality of the publications influences the quality of the database content. The analysis of randomly selected publications in SABIO-RK showed missing and inconsistent data. With 11% of the papers containing no temperature, 5% using ‘room temperature’, 4% containing no pH value and 3% no information about the buffer, outliers can also appear because of missing or inaccurate information in the publications (10). Users and curators can easily identify clusters or outliers of kinetic parameter values by navigating through the different visualizations.

Possible future steps to improve the visualization include increasing the number of operations that can be performed directly, i.e. locally on the graphs without the need to do a repeated search through the database, and removing the shown data number limit in the browser by implementing data clustering on the parallel coordinates graphs.

The new visualization is meant to support both modelers and experimentalists to extract the highest possible amount of information from accumulated and orderly presented data. Clustering and grouping of the data (e.g. kinetic parameters, EC numbers and environmental conditions) is implemented according to the needs of SABIO-RK users and curators. The visualization enables navigating through the database without the need to know much about available keywords in the database or about manually composing search queries, i.e. with minimal prior knowledge, thus making the data available for the wider user circle.

Moreover, the visual exploration and validation of SABIO-RK data help database curators to check the quality of database entries internally, before publishing data entries. For example, data extraction or annotation errors can occur based on typing errors, wrong annotations or imperfect curation work. Here, visualization can help to get a quick overview about the data and to facilitate the detection of curation errors. These internal quality checks will improve the reliability of the database and the user satisfaction.

## Data Availability

The data underlying this article are available in SABIO-RK database at https://sabiork.h-its.org and can be freely accessed.
